# Supplementary material for: Prognostic and Predictive Molecular Markers in Cholangiocarcinoma
Source: Cancers (Basel). 2022 Feb 17;14(4):1026. doi: 10.3390/cancers14041026 (PMC8870611; doi:10.3390/cancers14041026)
Supplement: Supplementary file 1 [file cancers-14-01026-s001.zip › cancers-1581306-supplementary.pdf]

Table S1. Overview of current and ongoing clinical trials for therapies targeting IDH1/2 in CCA.

Drug	Target	Indication	Clinical trial	Recruitment status / Results	Primary endpoint
**Isocitrate dehydrogenase 1/2 (IDH 1/2)**					
Ivosidenib (AG-120)	IDH1 inhibitor	Advanced CCA	NCT02989857 [150]	- Improved PFS<br>- Increased OS (when adjusted for crossover)<br>- Well safety and tolerability	PFS
		Advanced solid tumors including CCA	NCT02073994 (phase I)	Active, not recruiting	Safety/tolerability, MTD
		Advanced CCA	NCT04088188 (phase I)	Recruiting	DLT
Olutasidenib (FT-2102)	IDH1 inhibitor	Solid tumors including hepatobiliary tumors and iCCA	NCT03684811 (phase I/II)	Active, not recruiting	DLT, ORR
Enasidenib (AG-221)	IDH2 inhibitor	Advanced solid tumors including iCCA	NCT02273739 (phase I)	Completed	TEAE, DLT, ECOG status
Olaparib (AZD2281)	PARP inhibitor	Advanced solid tumors including CCA	NCT03212274 (phase II)	Recruiting	ORR
		Refractory or advanced CCA	NCT03878095 (phase II)	Recruiting	ORR
		Metastatic biliary tract cancer with Aberrant DNA repair gene mutations	NCT04042831 (phase II)	Recruiting	ORR
		Advanced biliary tract cancer	NCT04298021 (phase II)	Recruiting	DCR
		Advanced CCA	NCT04306367 (phase II)	Recruiting	ORR
		Solid tumors including CCA	NCT03991832 (phase II)	Recruiting	ORR, DCR
Dasatinib	IDH inhibitor	Advanced iCCA	NCT02428855 (phase II)	Completed	ORR

PFS: progression free survival; MTD: maximum tolerated dose; DLT: dose limiting toxicity; ORR: objective response rate; TEAE: treatment-emergent adverse events; ECOG: Eastern Cooperative Oncology Group; DCR: disease control rate; TTP: time to progression.

Table S2. Overview of current and ongoing clinical trials for therapies targeting KRAS in CCA.

Drug	Target	Indication	Clinical trial	Recruitment status / Results	Primary endpoint
**KRAS signaling pathway**					
Selumetinib (AZD6244)	MEK 1/2 inhibitor	Advanced biliary tract cancer	NCT00553332 (phase II) [153]	- Acceptable tolerability<br>- Promising anti-tumor activity	ORR
			NCT01242605 (phase I) [181]	- Modest efficacy<br>- Acceptable tolerability	Safety and tolerability
			NCT02151084 (phase II)	Active, not recruiting	ORR
Trametinib	MEK inhibitor	Advanced biliary cancer	NCT02042443 (phase II) [152]	Early termination due to no therapeutic response	OS
			NCT01943864 (phase II) [182]	- Well tolerated<br>- Primary endpoint not met	Non-progressive disease after 12 weeks
			NCT04566133 (phase II)	Recruiting	PFS
Dabrafenib	BRAF inhibitor	Multiple advanced tumors including CCA	NCT02034110 (phase II) [154]	- 51% investigator-assessed ORR<br>- 47% independent reviewer-assessed ORR<br>- Good tolerability	ORR
			NCT02465060 (phase II)	Recruiting	ORR
GSK1120212	MEK inhibitor	Advanced solid tumors, including CCA	NCT01438554 (phase I)	Completed	MTD, adverse events
Refametinib	MEK inhibitor	Advanced biliary tract cancer	NCT02346032 phase II)	Completed	ORR
MEK162	MEK inhibitor	Advanced biliary cancer	NCT01828034 (phase I/II)	Completed	MTD, PFS, ORR
		Advanced CCA	NCT04764084 (phase I)	Not yet recruiting	DLT
			NCT04895046 (phase II; recruiting)	Recruiting	PFS

PFS: progression free survival; OS: overall survival; MTD: maximum tolerated dose; DLT: dose limiting toxicity; ORR: objective response rate.

Table S3. Overview of current and ongoing clinical trials for therapies targeting FGFR in CCA.

Drug	Target	Indication	Clinical trial	Recruitment status / Results	Primary endpoint
		**Fibroblast growth factor (FGFR)**			
Infigratinib (BGJ398)	FGFR 1-3 kinase inhibitor	Advanced CCA	NCT02150967 (phase II) [113-115]	- Acceptable tolerability<br>- 14.8% ORR with 75.4% disease control rate	ORR
		Advanced CCA	NCT03773302 (phase III) [116]	Recruiting	PFS
		Advanced solid tumors including CCA	NCT04233567 (phase II)	Recruiting	ORR
Erdafitinib (JNJ-42756493)	Pan-FGFR kinase inhibitor	Advanced solid tumors including CCA	NCT01703481 (phase I) [108] (phase I)	Well tolerability	MTD
		Advanced NSCLC, urothelial cancer, esophageal cancer and CCA	NCT02699606 (phase II)	Active, not recruiting	ORR
Derazantinib (ARQ 087)	FGFR 1-3 kinase inhibitor	Advanced solid tumors including iCCA	NCT01752920 (phase I/II) [112]	- Well tolerability<br>- Anti-tumor activity	TEAE
		Advanced iCCA	NCT03230318 (phase II)	Recruiting	ORR, PFS
Futibatinib (TAS120)	FGFR 1-4 kinase inhibitor	Advanced solid tumors including CCA	NCT02052778 (phase II) [111]	- Efficacy in iCCA with FGFR2 aberrations<br>- Acceptable tolerability	Safety and RPTD, ORR
		Advanced or recurrent iCCA	NCT04093362 (phase III) [117]	Recruiting	PFS
CH5183284/Debio 1347	FGFR 1-3 kinase inhibitor	Advanced solid tumors including CCA	NCT01948297 (phase I) [103]	- Acceptable tolerability<br>- Preliminary efficacy	DLT, TEAE, TESAE
Pemigatinib (INCB054828)	FGFR 1-3 kinase inhibitor	Advanced or unresectable CCA	NCT02924376 [183] (phase II)	Preliminary efficacy	ORR
			NCT03656536 (phase III) [118]	Recruiting	PFS
			NCT04256980 (phase II)	Active, not recruiting	OSS
			NCT04088188 (phase I)	Recruiting	DLT
		Advanced malignancies including CCA	NCT02393248 (phase I/II)	Active, not recruiting	MTD, pharmacodynamics
E7090	FGFR 1-3 kinase inhibitor	Advanced CCA	NCT04238715 (phase II)	Recruiting	ORR

		Advanced CCA	NCT01752920 (phase I/II) [112]	Not yet recruiting	DLT
			NCT03230318 (phase II)	Recruiting	PFS

PFS: progression free survival; MTD: maximum tolerated dose; DLT: dose limiting toxicity; ORR: objective response rate; RPTD: recommended phase two dose; TEAE: treatment-emergent adverse events; TEASE: treatment-emergent serious adverse events. NSCLC: non-small cell lung cancer.

Table S4. Overview of current and ongoing clinical trials for therapies targeting EGFR and VEGF in CCA.

Drug	Target	Indication	Clinical trial	Recruitment status / Results	Primary endpoint
		**Epidermal growth factor (EGFR)**			
Erlotinib (Tarceva)	Selective EGFR inhibitor	Advanced CCA	NCT00350753 (phase II) [127]	- Efficient, good disease control rate<br>- Well tolerable	TTP, ORR
		Advanced CCA	NCT00356889 (phase II) [128]	- Anti-tumor activity<br>- Good tolerability	ORR
		Advanced biliary tract cancer	NCT00987766 (phase I) [184]	- Promising anti-tumor activity<br>- Acceptable tolerability	MTD, RPTD
		Advanced CCA, pancreatic cancer	NCT00266097 (phase I)	Completed	MTD
		Advanced biliary tract cancer, HCC	NCT00532441 (phase II) [185]	- Increased PFS<br>- OS comparable between erlotinib mono and erlotinib with doclitaxel	PFS
		Advanced biliary tract cancer	NCT01149122 (phase III) [135,186]	- No significant difference in PFS<br>- Early tumor shrinkage correlates with PFS	PFS
		Advanced CCA, gallbladder cancer	NCT01093222 (phase II)	Completed	PFS
		Advanced CCA, gallbladder cancer, HCC	NCT00033462 (phase II)	Completed	Rate of patients with PFS
		Metastatic biliary tract cancer	NCT03110484 (phase II)	Not yet recruiting	ORR
Lapatinib (GW572016)	Inhibitor of EGFR and Her-2/neu	Advanced CCA, HCC	NCT00101036 (phase II) [130]	- Well tolerability<br>- No clinical activity	ORR

			NCT00107536 (phase II) [131]	Terminated due to futility	ORR
Afatinib (BIBW 2992)	Inhibitor of EGFR and Her-2/neu	Advanced CCA	NCT01679405 (phase I) [187]	Terminated due to futility	TEAE
		Advanced solid tumors including CCA	NCT02451553 (phase I)	Active, not recruiting	TEAE, MTD, DLT, RPTD
Trastuzumab	Her-2/neu monoclonal antibody	Advanced CCA	NCT03613168 (phase II)[188]	Promising clinical activity	ORR, TEAS
		Advanced solid tumors including CCA	NCT02999672 (phase II);	Completed	ORR
		Advanced solid tumors including CCA	NCT04579380 (phase II)	Recruiting	ORR
		Advanced CCA	NCT04722133 (phase II)	Recruiting	ORR
		Advanced gastrointestinal cancers including CCA	NCT04430738 (phase I/II)	Recruiting	DLT, TEAS, incidence of laboratory abnormalities and dose alterations
		Advanced solid tumors including CCA	NCT02091141 (phase II) [137]	- Well tolerability<br>- Modest response rate	ORR
**Vascular endothelial growth factor (VEGF)**					
Bevacizumab	VEGF monoclonal antibody	Advanced CCA	NCT00350753 (phase II) [127]	- Efficient, good disease control rate<br>- Well tolerable	TTP, ORR
			NCT00356889 (phase II) [128]	- Anti-tumor activity<br>- Well tolerable	ORR
			NCT00361231 (phase II)	Competed	PFS
			NCT01206049 (phase II)	Completed	PFS
			NCT01007552 (phase II) [141]	No survival benefit compared to gemcitabine/cisplatin	PFS
			NCT04984980 (phase II)	Recruiting	Conversion rate
			NCT05052099 (phase Ib/II)	Recruiting	ORR
Ramucirumab	VEGFR2 monoclonal antibody	Advanced gastric or gastroesophageal junction adenocarcinoma, NSCLC, urothelium carcinoma, CCA	NCT02443324 (phase I) [189]	- Acceptable tolerability<br>- No improvement in OS	DLT

		Advanced CCA	NCT02520141 (phase II)	Active, not recruiting	PFS
			NCT02711553 (phase II)	Active, not recruiting	PFS
Apatinib	VEGFR2 inhibitor	Advanced iCCA	NCT03251443 (phase II) [190]	- Promising anti-tumor activity<br>- Acceptable tolerability	PFS, ORR, DCR
			NCT03521219 (phase II) [191]	- Improved ORR<br>- Acceptable tolerability	DCR
		Advanced CCA	NCT03427242 (phase II)	Recruiting	PFS
			NCT04642664 (phase II) [192]	- Promising anti-tumor activity<br>- Acceptable tolerability	PFS, ORR
			NCT03609489 (phase II)	Unknown	PFS
		Advanced iCCA	NCT04834674 (phase II)	Not yet recruiting	ORR, PFS
			NCT04454905 (phase II)	Recruiting	PFS

PFS: progression free survival; OS: overall survival; MTD: maximum tolerated dose; DLT: dose limiting toxicity; ORR: objective response rate; RPTD: recommended phase two dose; TEAE: treatment-emergent adverse events; DCR: disease control rate; TTP: time to progression.

Table S5. Overview of current and ongoing clinical trials for targeted therapies including multikinase inhibitors in CCA.

Drug	Target	Indication	Clinical trial	Recruitment status / Results	Primary endpoint
		**Multikinase inhibitors**			
Ponatinib	Multi-tyrosine kinase inhibitor	Advanced CCA	NCT02265341 (phase II)	Promising anti-tumor activity	ORR
		Solid tumors	NCT02272998 (phase II)	Recruiting	ORR
Pazopanib	Multi-kinase inhibitor	Advanced solid tumors including CCA	NCT01438554 (phase I) [109]	Terminated due to low response rate	MTD, TAES
		Advanced CCA	NCT01855724 (phase II) [193]	Terminated due to low response rate	ORR
Sorafenib	Multi-kinase inhibitor	Advanced CCA	NCT00919061 (phase II) [144]	No increase in efficacy compared to historical data	PFS
			NCT00661830 (phase II) [145]	No increase in efficacy compared to historical data	PFS
		Advanced CCA, gallbladder cancer	NCT01093222 (phase II)	Completed	PFS

		Advanced pancreatic and biliary carcinoma	NCT00634751 (phase I/II)	Completed	ORR
		Advanced CCA, gallbladder cancer	NCT00238212 (phase II)	Completed	ORR
		Advanced eCCA, gallbladder cancer	NCT02836847 (phase II)	Recruiting	PFS
			NCT03768375 (phase II)	Recruiting	PFS
Vandetanib	Multi-kinase inhibitor	Advanced CCA	NCT00753675 (phase II) [194]	No improvement in PFS	PFS
Cabozantinib	Multi-kinase inhibitor	Advanced CCA, HCC	NCT01954745 (phase II) [121]	- Limited activity<br>- Significant toxicity	PFS
			NCT02496208	Active, not recruiting	RPTD, TEAE
Regorafenib	Multi-kinase inhibitor	Advanced CCA	NCT02386397 (phase I/II) [195]	Acceptable tolerability	DLT
			NCT02053376 (phase II) [146]	Promising anti-tumor activity	PFS
			NCT02115542 (phase II) [147]	Modest clinical efficacy	OS
			NCT02162914 (phase II) [148]	Improvement in PFS and tumor control	PFS
		Solid tumors	NCT03475953 (phase I/II)	Recruiting	RPTD, ORR
Surufatinib	Multi-tyrosine kinase inhibitor	Advanced CCA	NCT02966821 (phase II) [196]	- Moderate clinical efficacy<br>- Tolerable safety profile	PFS
			NCT03873532 (phase II/III)	Recruiting	OS
			NCT05056116 (phase II)	Recruiting	ORR
			NCT05064852	Not yet recruiting	PFS
Lenvatinib	Multi-tyrosine kinase inhibitor	Advanced CCA	NCT02579616 [197] (phase II);	- Tolerable safety profile<br>-Anti-tumor activity	ORR
			NCT04656249 (phase II)	Completed	ORR, PFS
			NCT04211168 (phase II)	Recruiting	ORR, TEAE
		Solid tumors including CCA	NCT03797326 (phase II)	Active, not recruiting	ORR, TEAE
			NCT04976634 (phase II)	Recruiting	DLT, TEAE, ORR
		Unresectable iCCA	NCT04527679 (phase II)	Not yet recruiting	ORR
		Advanced CCA	NCT04550624 (phase II)	Recruiting	ORR

		Advanced iCCA	NCT04361331 (phase II)	Active, not recruiting	ORR
			NCT03951597 (phase II);	Active, not recruiting	ORR
		Advanced CCA	NCT03895970 (phase II)	Recruiting	ORR, DCR, PFS
Larotrectinib	Selective tyrosin kinase inhibitor of tropomysin receptor kinases	Solid tumors including CCA with NTRK gene fusion	NCT02576431 (phase II)	Recruiting	ORR
Entrectinib	Selective tyrosin kinase inhibitor of tropomysin receptor kinases	Solid tumors including CCA with NTRK gene fusion	NCT02568267 (phase II)	Recruiting	ORR

PFS: progression free survival; OS: overall survival; MTD: maximum tolerated dose; DLT: dose limiting toxicity; ORR: objective response rate; RPTD: recommended phase two dose; TEAE: treatment-emergent adverse events; DCR: disease control rate; TTP: time to progression.

Table S6. Overview of current and ongoing clinical trials for therapies targeting the MET, notch, and chromatin remodeling pathways in CCA.

Drug	Target	Indication	Clinical trial	Recruitment status / Results	Primary endpoint
		**MET signaling pathway**			
Tivantinib (ARQ 197)	Selective c-MET inhibitor	Advanced solid tumors including CCA	NCT00874042 (phase I) [120]	Improved response or stable disease in combination with gemcitabine	MTD, RPTD
Merestinib	MET inhibitor	Advanced CCA	NCT03027284 (phase I) [198]	Well tolerability	DLT
			NCT02711553 (phase II)	Active, not recruiting	PFS
		**Chromatin remodeling**			
Niraparib	PARP1 inhibitor	CCA, renal cell carcinoma, uveal melanoma, mesothelioma	NCT03207347 (phase II)	Active, not recruiting	ORR
		Multiple carcinomas including CCA	NCT04779151 (phase II)	Not yet recruiting	ORR
		Solid tumors including CCA	NCT04178460 (phase I)	Recruiting	DLT, MTD, RPTD, ORR
		Advanced CCA	NCT04764084 (phase I)	Not yet recruiting	DLT
			NCT04895046 (phase II; recruiting)	Recruiting	PFS
		**Notch signaling pathway**			
CB-103	pan-Notch inhibitor	Advanced solid tumors including CCA	NCT03422679 (phase I/II)	Recruiting	DLT, ORR

PFS: progression free survival; MTD: maximum tolerated dose; DLT: dose limiting toxicity; ORR: objective response rate; RPTD: recommended phase two dose.

Table S7. Overview of current and ongoing clinical trials for immunotherapies in CCA

Drug	Target	Indication	Clinical trial	Recruitment status / Results	Primary endpoint
**Immunotherapy**					
M7824 (Bintrafusp Alfa)	Fusion protein targeting PD-L1 and TGF-β	Advanced CCA	NCT02699515 (phase I)	Active, not recruiting	DLT, TEAE
			NCT03833661 (phase II)	Active, not recruiting	ORR
			NCT04066491 (phase II/III)	Active, not recruiting	DLT, OS
		Advanced iCCA	NCT04708067 (phase I)	Recruiting	TEAE
		Advanced CCA	NCT04727541 (phase II)	Recruiting	Major pathologic Response (MPR)
Atezolizumab	PD-L1 antibody	Advanced CCA	NCT04677504 (phase II) [199]	Active, not recruiting	PFS
Pembrolizumab	PD-1 antibody	Advanced CCA	NCT02703714 (phase II)	Completed	ORR
			NCT04003636 (phase III)	Active, not recruiting	OS
		Advanced solid tumors including CCA	NCT02628067 (phase II) [157]	- 40.9% ORR in a subset of patients with CCA<br>- Good tolerability	ORR
Nivolumab	PD-1 antibody	Multiple tumors including CCA	NCT02834013 (phase II)	Recruiting	ORR
Durvalumab	PD-L1 antibody	Advanced CCA	NCT03875235 (phase III)	Completed	OS

PFS: progression free survival; OS: overall survival; DLT: dose limiting toxicity; ORR: objective response rate; TEAE: treatment-emergent adverse events.
